# Effects of Four Isothiocyanates in Dissolved and Gaseous States on the Growth and Aflatoxin Production of *Aspergillus flavus* In Vitro

**DOI:** 10.3390/toxins14110756

**Published:** 2022-11-02

**Authors:** Yohei Hareyama, Mitsunori Tarao, Koki Toyota, Tomohiro Furukawa, Yoshiharu Fujii, Masayo Kushiro

**Affiliations:** 1 Department of Food Energy System Science, Graduate School of Bio-Application and System Engineering Sciences, Tokyo University of Agriculture and Technology, 2-24-16, Tokyo 184-8588, Japan; 2 Institute of Food Research, National Agriculture and Food Research Organization (NARO), 2-1-12 Kannondai, Tsukuba 305-8642, Japan; 3 Faculty of Agriculture, Tokyo University of Agriculture and Technology, Tokyo 183-8506, Japan

**Keywords:** mycotoxin, antifungal activity, benzyl isothiocyanate, phenylethyl isothiocyanate, methyl isothiocyanate, botanical compound, state of matter

## Abstract

Aflatoxins (AFs), a class of toxins produced by certain species of the genus *Aspergillus*, occasionally contaminate food and cause serious damage to human health and the economy. AFs contamination is a global problem, and there is a need to develop effective strategies to control aflatoxigenic fungi. In this study, we focused on isothiocyanates (ITCs) as potential chemical agents for the control of aflatoxigenic fungi. We quantitatively evaluated the effects of four ITCs (allyl ITC (AITC), benzyl ITC (BITC), and methyl and phenylethyl ITCs) in dissolved and gaseous states on the growth and aflatoxin B_1_ production of *Aspergillus flavus*. In experiments using dissolved ITCs, BITC was found to be the strongest inhibitor of growth and aflatoxin B_1_ production by *A. flavus*. Meanwhile, in the gaseous state, AITC strongly inhibited the *A. flavus* growth. When the concentration of ITCs in the liquid medium was quantified over time, AITC levels decreased to below the detection limit within 24 h, whereas BITC levels remained stable even after 48 h. These results suggested that when ITCs are utilized to control aflatoxigenic fungi, it is necessary to use them in a dissolved or gaseous state, depending on their volatility.

## 1. Introduction

Aflatoxins (AFs) are fungal secondary metabolites mainly produced by certain strains of *Aspergillus flavus* and *A. parasiticus*. AFs are extremely toxic to humans and livestock and are classified as group 1 carcinogens to humans by the International Agency for Research on Cancer [1]. Contamination of food and feed with AFs is a global problem in terms of food and feed safety and security, especially in developing countries. In Kenya, 125 people died in 2004 due to mass food poisoning caused by the acute toxicity of AFs [2]. Globally, approximately 155,000 people per year are estimated to have hepatocellular carcinoma due to potential AFs exposure worldwide [3]. Furthermore, AFs contamination causes enormous economic losses because AFs are chemically stable and are difficult to remove or deconstruct from contaminated food materials [4]. Lubulwa et al. estimated the total social cost of AFs contamination in Indonesia, the Philippines, and Thailand in 1991 to be USD 476.9 million [5]. Mitchell et al. estimated that in climate-change-susceptible years, the economic loss of corn from AFs in the U.S. could be more than USD 1 billion [6].

Various strategies have been investigated to reduce the damage caused by AFs contamination. Biological pesticides using atoxigenic strains of aflatoxin are an effective method for AFs control and have been put to practical use in several countries [7]. This reduces AFs contamination by causing competition with toxigenic strains. Adsorbents to prevent AFs absorption in vivo have also been studied [8]. Experiments on the dynamic gastrointestinal-tract-simulated model conducted by Vázquez-Durán et al. showed that plant-derived adsorbents have the potential to remove AFB_1_ (up to 93.6%). Efforts to reduce aflatoxin-induced damage in vivo have been reported by Jin et al. [9]. Their study shows that curcumin from turmeric rhizomes protects the ileum from acute damage caused by AFB_1_ administration in ducks. However, to fundamentally prevent the AFs contamination, it is necessary to control the aflatoxigenic fungi.

Previously, many kinds of inhibitors of growth and AFs production of aflatoxigenic fungi have been explored [10]. Isothiocyanates (ITCs), which are sulfur-containing organic compounds mainly derived from *Brassicaceae* plants, inhibit the growth of various harmful microorganisms [11]. We had reported that methyl isothiocyanate (MITC), a soil and wood fumigant, inhibited the growth and aflatoxin B_1_ (AFB₁) production of *A. flavus* more strongly than allyl isothiocyanate (AITC), a pungent component of horseradish, in a liquid-medium assay [12]. Saladino et al. reported the antifungal activities of AITC, benzyl isothiocyanate (BITC), and phenyl isothiocyanate (PITC) against *A. parasiticus* [13]. Okano et al. investigated the inhibitory effects of gaseous AITC on *A. parasiticus* inoculated on corn and peanuts [14,15]. Although some studies have reported the antifungal activities of ITCs against *A. parasiticus*, quantitative and comparative information on the antifungal activities of ITCs against *A. flavus* is lacking. Since *A. flavus* is a major AFs producer and the most important fungus in AFs control, it is necessary to investigate the antifungal activities of ITCs on *A. flavus* for AFs control [16,17].

**Figure 1:**
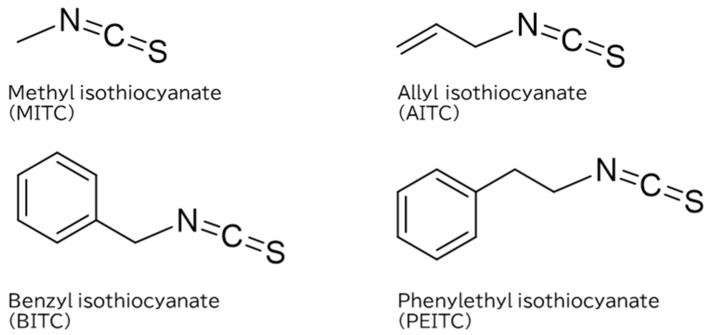
Chemical structures of isothiocyanates (ITCs) used in the experiments.

In this study, to identify effective ITCs for controlling AFs contamination, we evaluated the inhibitory activities of four ITCs (AITC, MITC, BITC, and phenylethyl isothiocyanate (PEITC)) (Figure 1), on AFB_1_ production and the growth of *A. flavus*. The reason we choose PEITC in this study is because it exerts inhibitory effects on *Fusarium graminearum*, *Alternaria alternata*, *Escherichia coli*, and various other microorganisms [18,19,20] but not on aflatoxigenic fungi.

As ITCs are highly volatile, aflatoxigenic fungi may be controlled using volatilized ITCs as fumigants. However, Saladino et al. reported that the intensity of antifungal activity differed depending on the state of ITCs [11]. Therefore, in addition to assays of the dissolved state of ITCs, the effects of gaseous ITCs on *A. flavus* were investigated in this study.

## 2. Results

### 2.1. Antifungal Activity and Time-Course Action of Dissolved ITCs

**Figure 2:**
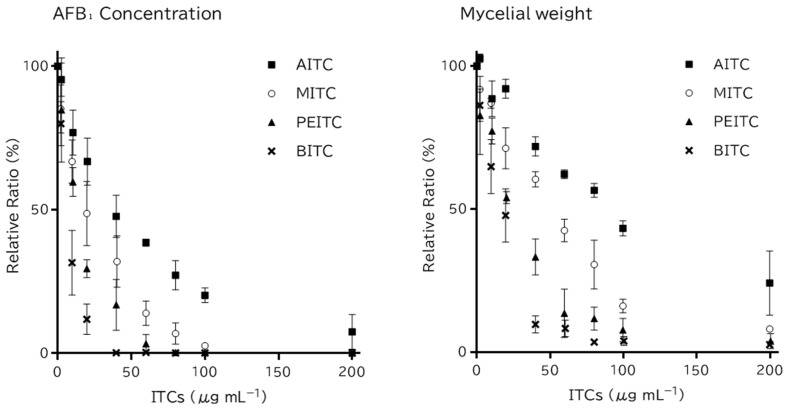
Relative ratio of Aflatoxin B_1_ (AFB_1_) concentration (**left**) and mycelial weight (**right**) of *Aspergillus flavus* MAFF 111229 as compared with 0 μg mL^−1^ treatment at each concentration of isothiocyanates (ITCs). Error bar indicates the standard error of the mean of three independent experiments (each in triplicate).

The effects of dissolved ITCs on the *A. flavus* strain MAFF 111229 were compared in a liquid culture medium. All ITCs decreased the AFB₁ concentration in the medium and the mycelial weight of *A. flavus* in a dose-dependent manner (Table 1, Figure 2). BITC showed the strongest activity and decreased both the AFB₁ concentration and mycelial weight below the lower limit of quantification at 40 µg mL^−1^. PEITC showed the second-highest inhibitory activity, followed by MITC, whereas AITC had the lowest inhibitory activity. The half-maximal inhibitory concentration (IC_50_) values of AITC, BITC, MITC, and PEITC for AFB₁ production were estimated to be 34.7, 5.4, 17.4, and 11.6 μg mL^−1^, respectively. The IC_50_ values of AITC, BITC, MITC, and PEITC for the growth of *A. flavus* were 86.2, 15.2, 43.8, and 21.4 µg mL^−1^, respectively.

The concentration of ITCs added to the liquid culture medium at an initial concentration of 200 μg mL^−1^ was quantified after 0.5, 24, and 48 h (Table 2). AITC and MITC were not detected after 24 h, whereas BITC and PEITC were detected even after 48 h.

### 2.2. Antifungal Activities of Gaseous ITCs

**Figure 3:**
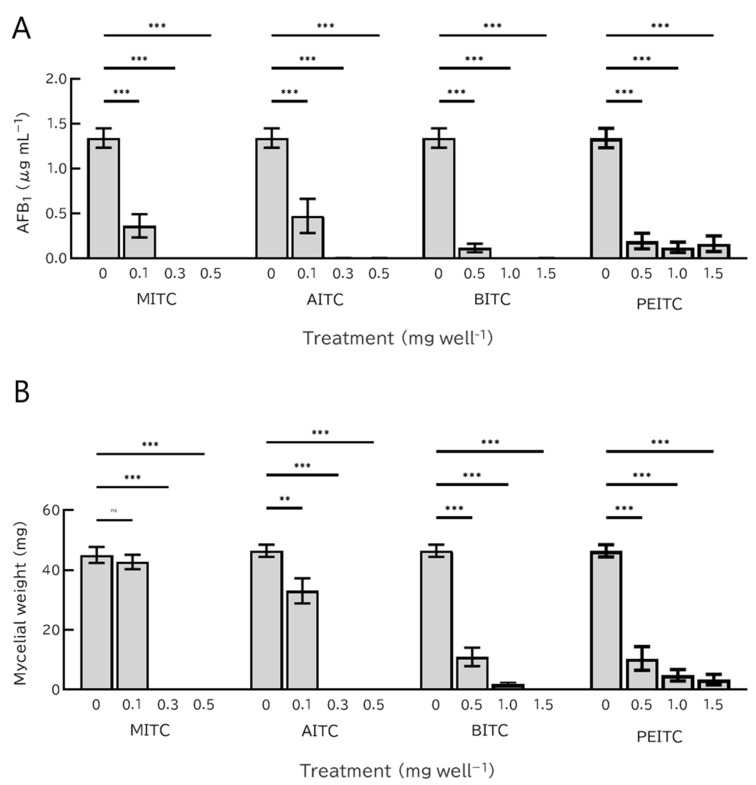
AFB_1_ concentration in the medium (**A**) and dry weight of *A. flavus* (**B**) after four days of incubation with gaseous isothiocyanates (ITCs). Each experiment was performed in triplicate, and error bars indicate the standard error. Differences between the control and each treatment were analyzed using one-way analysis of variance (ANOVA) followed by Dunnett test; ns = not significant, ** *p* < 0.01, *** *p* < 0.001.

The effects of gaseous ITCs on the *A. flavus* strain MAFF 111229 were compared using the modified dish pack method. In contrast to the dissolved state, AITC and MITC showed the strongest inhibitory effects in the gaseous state, followed by BITC, whereas PEITC showed the lowest inhibitory effect (Figure 3). When 0.1 mg well^−1^ of AITC or MITC was added, the AFB₁ concentration in the medium was significantly reduced (*p* < 0.001) (Figure 3). The addition of 0.5 mg well^−1^ of BITC or PEITC also reduced the AFB_1_ concentration (*p* < 0.001). However, even 1.5 mg well^−1^ PEITC could not completely inhibit AFB_1_ production.

Mycelial growth decreased to below the detection limit when 0.3 mg of MITC or AITC was added (Figure 3). However, only AITC significantly reduced mycelial growth at 0.1 mg (*p* < 0.05). In BITC and PEITC treatments, 0.5 mg of BITC and PEITC decreased the mycelial growth (*p* < 0.001). Unlike the other three ITCs, adding 1.5 mg of PEITC did not completely inhibit the growth of *A. flavus*.

### 2.3. Effects of Short-Term ITC Exposure on the Growth of A. flavus

*A. flavus* spores were exposed to ITCs in YES liquid medium for 3 and 24 h, and then the cultures were inoculated onto ITCs-free GY agar medium and incubated for four days. The effects of short-term exposure to dissolved ITCs for 3 and 24 h on *A. flavus* were investigated. Among the ITCs, the strongest activity was observed with MITC, which reduced fungal growth by exposing at the concentrations of 100 µg mL^−1^ or higher for at least 3 h (Table 3). Growth was completely inhibited by exposing at the concentrations of 150 µg mL^−1^ or higher for 24 h. Similar to MITC, AITC was the second-most active ITC that inhibited fungal growth by exposing at the concentrations of 150 µg mL^−1^ or higher for 24 h. Although the inhibitory activity of 3 h exposure to BITC was similar to that of AITC at a concentration of 200 μg mL^−1^, BITC was required 24 h of exposure to completely inhibit the growth of *A. flavus*. PEITC had no effect on growth by 3 h of exposure, whereas fungal growth was observed by 24 h of exposure at all concentrations.

## 3. Discussion

The ITC that most strongly inhibited the growth and AFB_1_ production of *A. flavus* differed between the dissolved and gaseous state. In the dissolved states, BITC showed the strongest antifungal activity, whereas in the gaseous state, AITC and MITC more strongly inhibited the growth and AFB_1_ production of *A. flavus.* Saladino et al. reported a similar trend in their results [13]. They investigated the antifungal activities of AITC, BITC, and PITC in the dissolved and gaseous states against *A. parasiticus*. In their experiments, BITC showed the strongest antifungal activity after 72 h of exposure in a liquid culture medium, whereas AITC inhibited the colony formation of *A. parasiticus* most strongly in the gaseous state. Kurt et al. investigated the effects of seven ITCs, including dissolved and gaseous AITC, BITC, MITC, and PEITC, on *Sclerotinia sclerotiorum* [21]. Their report showed a trend similar to ours, with BITC showing the strongest antifungal activity in the dissolved state and MITC inhibiting mycelial growth, most notably in the gaseous state. ITCs are highly volatile and are thought to gradually disappear from the liquid medium owing to volatilization. Therefore, we hypothesized that the volatility of each ITC is involved in the differences in the intensities of their antifungal activity between dissolved and gaseous states. To verify this, we examined the time-course changes in the remaining ITCs in the liquid culture medium and found that AITC and MITC disappeared from the medium faster than BITC and PEITC (Table 2). These results mean that MITC and AITC only affected the *A. flavus* before it disappeared from the medium, whereas BITC and PEITC remained in medium and maintained their antifungal efficacy against *A. flavus* for a longer time than MITC and AITC. In short-term exposure experiments, MITC and AITC inhibited fungal growth slightly more strongly than BITC and PEITC after 24 h of exposure (Table 3). These results support the consideration that BITC and PEITC affect for a longer time than MITC and AITC, resulting in stronger antifungal activity. Ashiq et al. also reported that 16–17 h of exposure to AITC and MITC in a liquid culture medium inhibited conidial germination in *F. graminearum* more strongly than BITC and PEITC [16]. Based on these results, the antimicrobial activity observed in the liquid culture medium assay is not only related to the intensity of the antimicrobial activity of the substance but also to its residual time in the medium.

In the gaseous state, the susceptibility of *A. flavus* to ITCs was similar to that of the other microorganisms. Azaiez et al. reported that gaseous AITC inhibited mycelial growth of *F. oxysporum*, a fumonisin-producing fungus, more potently than BITC [22]. Yang et al. also investigated the inhibitory effects of gaseous AITC and BITC on *A. niger*, *A. carbonarius*, and *A. ochraceus* and calculated their respective IC_50_ values [23]. These reports are consistent with our results that gaseous AITC inhibits fungal growth more strongly than BITC. On the other hand, Kara et al. studied the antifungal activities of gaseous MITC, AITC, BITC, PEITC, EITC, and butyl ITC against *Geotrichum citri-aurantii* and revealed that BITC inhibited germination and mycelial growth at the lowest concentration [24]. Taylor et al. examined the effects of seven dissolved ITCs against *Colletotrichum coccodes*, *Rhizoctonia solani,* and *Helminthosporium solani* [25]. They reported that PEITC was the strongest inhibitor of *C. coccodes* and *H. solani*, while MITC and BITC were most effective against *R. solani*. Therefore, the susceptibility of microorganisms to ITCs is not always consistent.

Both mycelial growth and AFB_1_ production decreased in almost all the treatments. ITCs are presumed to react nonspecifically with random proteins due to the high reactivity of the ITCs group (-N=C=S) [26]. Therefore, the antifungal activity of ITCs against *A. flavus* is also likely due to a nonspecific reaction. In contrast, AFB_1_ production was affected by ITCs before mycelial growth in most of the treatments. Furukawa et al. reported that mitochondrial energy metabolism indirectly regulates AFs production of *A. flavus* [27]. Namely. this suggests that AFs production may be regulated by primary metabolism. Thus, it is possible that the inhibition of mycelial growth resulted due to a decrease in AFB_1_ production.

In Table 2, the recovery rate for this extraction method was 70–85%. Thus, the actual concentration of ITCs in the medium was considered higher than measured value. The decrease in the concentration of ITCs over time means a decrease in toxicity to humans but also a loss of antifungal activity. However, as can be seen from Table 3, even short-term exposure to ITCs showed effective inhibition. In particular, exposure to MITC above 150 µg mL^−1^ and AITC or BITC above 200 µg mL^−1^ for 24 h each showed fungicidal activity. Therefore, it is expected that when ITCs are actually applied to AFs control, they will show effective fungicidal activity for a short time and then volatilize, resulting in less persistence in the food. 

Based on the results, we conclude that this study showed the potential for ITCs to be applied to control *A. flavus*. However, the most practical ITC for AFs control differs, depending on the state of the compound. Thus, for example, AITC and MITC are both suitable fumigants; however, BITC and PEITC are more practical in terms of residual effects. 

## 4. Conclusions

The antifungal activities of ITCs against *A. flavus* differed between the dissolved and gaseous states, depending on the volatility and antifungal activity of the compound. Based on these results, suitable ITCs should be selected for AFs control depending on their potential use and purpose.

## 5. Materials and Methods

### 5.1. Chemicals

AF reagents (AFB_1_, AFB_2_, AFG_1_, AFG_2_ mixture solution, 25 µg mL^−1^) for mycotoxin assay, AITC, ethanol, methanol, glucose, and hexane were purchased from FUJIFILM Wako Pure Chemical Corporation (Osaka, Japan). MITC, BITC, and trifluoroacetic acid were purchased from Tokyo Chemical Industry Co., Ltd. (Tokyo, Japan). PEITC was purchased from Acros Organics (Geel, Belgium). Acetonitrile and sucrose were purchased from NACALAI TESQUE, Inc. (Kyoto, Japan). Potato dextrose broth, yeast extract, and agar were purchased from BD Biosciences (Franklin Lakes, NJ, USA). Ethyl acetate was purchased from KISHIDA CHEMICAL Co., Ltd. (Osaka, Japan).

### 5.2. A. flavus Isolate

*A. flavus* strain MAFF 111229 obtained from the NARO Genebank (Tsukuba, Japan) was used for all experiments. *A. flavus* was cultivated on potato dextrose agar medium at 25 °C for a week. After cultivation, the surface of the medium was rinsed with 0.05% Tween 80, and the crude suspension was filtered through a Mira cloth (Merck, Darmstadt, Germany) to obtain the spore suspension.

### 5.3. Antifungal Assay for Dissolved ITCs

The antifungal activities of the dissolved ITCs were determined using the tip culture method [28]. First, 1 mL tip of micropipette was stuffed with quartz wool. The tip was set in a 5 mL glass tube, and covered with an aluminum cap. After the tube was autoclaved, the end of the tip was sealed with Parafilm. Amounts of 244 µL of YES medium (20% sucrose and 2% yeast extract), 5 µL of spore suspension (4.3 × 10^6^ spores per mL), and 1 µL of ITC solution were mixed, and a total of 250 µL of mixture was added to the tip. The ITCs were diluted with ethanol, and the final concentration was adjusted to 2, 10, 20, 40, 60, 80, 100, and 200 µg mL^−1^. The control was prepared by adding ethanol and was used to evaluate the antifungal activities of ITCs. It has been confirmed that this concentration of ethanol (0.4%) does not affect the growth and AFs production of *A. flavus* (date not shown). Cultivation was conducted at 25 °C for five days in the dark in a metallic container with distilled water to maintain humidity. After cultivation, the mycelia and medium were separated by centrifugation. The fresh weight of mycelia was determined to evaluate the growth of *A. flavus*. The medium was used to evaluate AFB_1_ production by *A. flavus* according to the method described by Kushiro et al. [29]. A portion of the medium was evaporated and treated with trifluoroacetic acid to convert AFB₁ into the highly fluorescent hemiacetal AFB_2a_, which was analyzed using high-performance liquid chromatography (HPLC).

### 5.4. Antifungal Assay for Gaseous ITCs

**Figure 4:**
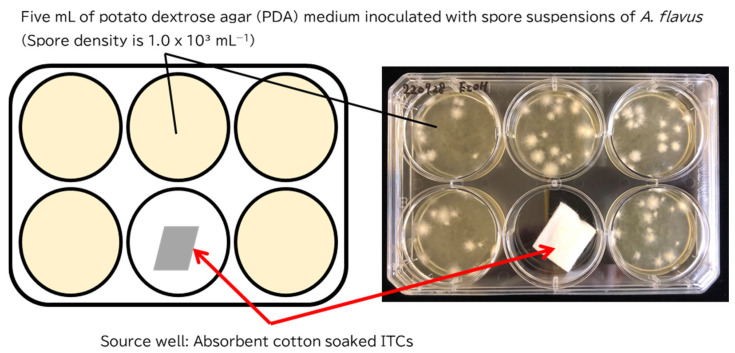
Modified dish pack method for measuring the antifungal activities of volatile components.

The antifungal activities of gaseous ITCs were determined using the dish pack method with some modifications (Figure 4) [30]. Multi-well plastic dishes with six wells (Thermo Fisher Scientific, Waltham, MA, USA) were used for this experiment. Five of the six wells were used to culture *A. flavus.* The remaining well contained the source of volatiles and was referred to as the source well. Five milliliters of PDB medium inoculated with *A. flavus* to a spore density of 1.0 × 10^3^ spores mL^−1^ was added to each of the five wells. The source well was placed in 0.1 g of cotton soaked in 100 µL of an ethanol solution of the ITCs. The concentrations of the ethanol solution of AITC and MITC were 1, 3, or 5 mg mL^−1^. The concentrations of the ethanol solution of BITC and PEITC were 5, 10, or 15 mg mL^−1^. Thus, the final amount of MITC and AITC per well were 0.1, 0.3, or 0.5 mg well^−1^ and those of BITC or PEITC were 0.5, 1.0, or 1.5 mg well^−1^. The control was prepared by adding ethanol and was used to evaluate the antifungal activities of ITCs. It has been confirmed that this amount of ethanol does not affect the growth and AFs production of *A. flavus* (date not shown). Each dish was sealed on the sides with cellophane tape to avoid leakage of ITCs. Cultivation was conducted at 25 °C in the dark for four days. After cultivation, cultures from three wells adjacent to the source well were transferred to a 15-mL tube and centrifuged at 400× *g* for 5 min to divide the mycelia and medium. A part of the medium was taken in a different test tube and analyzed for AFB₁ concentration using the same method as that described in Section 5.3. The mycelia were freeze-dried and weighed to evaluate their growth.

### 5.5. HPLC with Fluorescence Detection (HPLC-FL) for the Quantification of AFB_1_

The analytical conditions for AFB_1_ were as described by Kushiro et al. [29]. The HPLC-FL system was composed of an HPLC column Capcell pack C18 UG120 (5 μm, 250 mm × 4.6 mm i.d.; Osaka Soda, Osaka, Japan), pump LC-20AD, column heater CTO-10A, autosampler SIL-20AC, fluorescence detector RF-20A, communication bus module CBM-20A, and LabSolutions software (Shimadzu, Kyoto, Japan). The mobile phase consisted of water, methanol, and acetonitrile (6:3:1), and the flow rate was 0.3 mL min^−1^. The column heater temperature was set at 40 °C. AFB_1_ was detected at wavelengths of 365 (extraction) and 450 nm (emission). The standard AF mix was diluted to generate the calibration curve. The limit of detection (LOD) and limit of quantitation (LOQ) for the analysis were approximately 0.25 and 0.74 ng mL^−1^, respectively, as calculated from the standard deviation of the y-intercept on the calibration curve.

### 5.6. Quantification of ITC Concentration in the Medium over Time

The concentrations of ITCs in YES liquid medium were quantified over time using the wine extraction method with some modifications [31]. ITC solution was added to the YES liquid medium, and the concentration was adjusted to 200 μg mL^−1^. After 0.5, 24, and 48 h, ITCs were extracted from the medium and quantified using HPLC or gas chromatography (GC). MITC and AITC were extracted via liquid–liquid partitioning with acetonitrile and analyzed via HPLC. Briefly, 200 μL of the YES liquid medium containing ITCs was taken in a 2 mL tube, and 200 μL of acetonitrile was added and mixed. Subsequently, 150 μL of acetonitrile layer was transferred to another tube. This procedure was repeated two more times and obtain a total of 550 μL of acetonitrile extract. This acetonitrile extraction was analyzed by HPLC. BITC and PEITC were concentrated in a solid-phase extraction column and analyzed using GC. A pre-conditioned C18 solid-phase extraction column (InertSep C18, 5 mg per 300 mL; GL Sciences Inc., Tokyo, Japan) was filled with 400 µL medium. After washing with 1 mL of 10% methanol, the column was eluted with 1 or 2 mL of ethyl acetate and used for analysis via GC.

#### 5.6.1. HPLC with Diode Array Detection for the Quantification of MITC and AITC

MITC and AITC were analyzed using an Agilent HPLC 1100 series (pump G1311A, Degasser G1322A, column heater G1316A, autosampler G1316A, diode array detector G1315A, multichannel interface 35900E, and OpenLab CDS ChemStation Edition for LC & LC/MS System; Agilent Technologies, Inc., Santa Clara, CA, USA) equipped with a 5C18-MS-Ⅱ packed column (5 μm, 250 mm × 4.6 mm, i.e., Nacalai Tesque, Kyoto, Japan). The analytical conditions for MITC and AITC were determined by HPLC, using the method described by Abe [32]. The mobile phase was 60% acetonitrile at a flow rate of 1 mL min^−1^. The column heater was maintained at 30 °C. The AITC and MITC were measured at a wavelength of 240 nm. Calibration curves were prepared by diluting MITC and AITC with acetonitrile. The instrumental LOD and LOQ of MITC and AITC were 0.2 and 0.6 μg mL^−1^, respectively. 

#### 5.6.2. GC with Flame Ionization Detection (GC-FID) for the Quantification of BITC and PEITC

GC-FID analysis was performed using a GC-2010 instrument (Shimadzu, Kyoto, Japan) equipped with a capillary column (DB-WAX, 30 m × 0.32 mm i.d., 0.5 μm film thickness; Agilent Technologies, Inc., Santa Clara, CA, USA), an autosampler (AOC-20i; Shimadzu, Kyoto, Japan), an air compressor (Nishishiba Electric Co., Ltd., Kobe, Japan), and LabSolutions software (Shimadzu, Kyoto, Japan). Helium (grade 1, >99.99995 vol%; Taiyo Nippon Sanso Corporation, Tokyo, Japan) was used as the carrier gas, with a column flow rate of 0.98 mL min^−1^. The injector temperature was 240 °C, and all injections were performed in a 1:10 split. The GC oven temperature was maintained at 220 °C for 15 min. The detector temperature was maintained at 240 °C, and the flow rates were 40 mL min^−1^ for hydrogen, 30 mL min^−1^ for makeup gas (helium), and 400 mL min^−1^ for air. BITC and PEITC were diluted with ethyl acetate to generate calibration curves. The LOD and LOQ of BITC were approximately 1.55 and 4.70 μg mL^−1^, respectively. The LOD and LOQ of PEITC were approximately 1.46 and 4.44 μg mL^−1^, respectively. The LOD and LOQ values were calculated from the standard deviation of the estimated blank response values.

### 5.7. Effects of Short-Term ITC Exposure on the Germination and Growth of A. flavus

ITC solution was then added to the YES liquid medium. The concentrations of the ITCs were adjusted to 50, 100, 150, and 200 μg mL^−1^. A spore suspension of *A. flavus* was added to the medium to adjust the final density to 1.0 × 10^3^ mL^−1^. The suspension was then incubated at 25 °C in the dark for 24 h. After 24 h of incubation, a portion of the culture was inoculated onto an ITC-free GY agar medium (2% glucose, 0.5% yeast extract, and 2% agar) and incubated at 25 °C in the dark for four days. After incubation, the emerging colonies were observed, and their effects on the growth of *A. flavus* were evaluated.

### 5.8. Statistical Analysis

The antifungal activity assay for both liquid and gaseous ITCs was performed in triplicate for each treatment, and each treatment was repeated three times. The data represent the mean and standard error calculated from the average of each experiment. Differences between treatments were analyzed using one-way analysis of variance, followed by Dunnett’s test. The IC_50_ value was calculated using the statistical analysis software GraphPad Prism version 9.1.2 (Dotmatics, Boston, MA, USA). The experimental data measuring the concentrations of ITCs represent the mean and standard deviation of the data obtained from three replicates.

## Figures and Tables

**Table 1:** Aflatoxin B_1_ (AFB_1_) concentration (μg mL^−1^) in the medium, mycelial fresh weight (mg), and half-maximal inhibitory concentration (IC_50_) values after the exposure of *Aspergillus flavus* MAFF 111229 to isothiocyanates (ITCs) via the tip culture method.

ITCs		Concentration of Exposed ITCs (μg mL^−1^)								
		0	2	10	20	40	60	80	100	200
AITC	AFB_1_	27.2 ± 0.5	25.9 ± 1.1	20.8 ± 1.7	18.1 ± 2.0	12.9 ± 1.8	10.5 ± 0.4	7.4 ± 1.4	5.5 ± 0.7	2.0 ± 1.6
	Mycelial weight	24.4 ± 2.6	25.0 ± 2.4	21.9 ± 3.6	22.5 ± 2.6	17.6 ± 2.3	15.2 ± 1.8	13.8 ± 1.5	10.7 ± 1.5	<LOQ
BITC	AFB_1_	31.2 ± 0.6	25.0 ± 2.9	9.9 ± 3.6	3.7 ± 1.6	<LOQ	<LOQ	<LOD	<LOD	<LOD
	Mycelial weight	28.8 ± 0.6	24.9 ± 1.9	18.7 ± 3.0	13.8 ± 2.8	<LOQ	<LOQ	<LOD	<LOD	<LOD
MITC	AFB_1_	31.6 ± 0.5	27.0 ± 3.0	21.1 ± 2.5	15.5 ± 3.7	10.1 ± 2.9	4.4 ± 1.4	2.2 ± 1.2	0.8 ± 0.4	<LOD
	Mycelial weight	28.3 ± 0.2	26.0 ± 0.4	24.6 ± 0.6	20.2 ± 2.1	17.1 ± 0.9	12.0 ± 1.2	8.7 ± 2.5	<LOQ	<LOQ
PEITC	AFB_1_	27.2 ± 0.5	23.0 ± 4.6	16.2 ± 1.1	8.0 ± 2.8	4.6 ± 2.4	0.9 ± 0.9	<LOQ	<LOQ	<LOD
	Mycelial weight	24.4 ± 2.6	19.7 ± 3.1	19.0 ± 2.9	13.3 ± 1.8	7.9 ± 2.1	<LOQ	<LOQ	<LOD	<LOD

Experiments were performed in triplicate and the standard error of each mean value is shown. LOD and LOQ indicate the limit of detection and limit of quantitation, respectively. LOD and LOQ of AFB_1_ were 0.06 and 0.18 μg mL^−1^, respectively. LOD and LOQ of mycelial weight were 1.95 and 3.95 mg, respectively.

**Table 2:** Measured value of remaining ITCs concentration (μg mL^−1^) in the liquid culture medium when ITCs were added to 200 μg mL^−1^ over time.

	Hour		
	0.5	24	48
AITC	140.6 ± 3.4	<LOD	<LOD
BITC	153.6 ± 9.7	12.3 ± 4.3	7.9 ± 3.1
MITC	169.2 ± 13.0	<LOD	<LOD
PEITC	154.1 ± 15.7	10.8 ± 2.1	7.2 ± 3.6

Mean values and standard deviations are shown (*n* = 3). The limit of detection (LOD) for both methyl isothiocyanate (MITC) and allyl isothiocyanate (AITC) was 0.6 μg mL^−1^.

**Table 3:** Growth of *A. flavus* after short-term exposure to isothiocyanates (ITCs).

ITCs (μg mL^−1^)		Exposure Time (Hour)		
		0	3	24
AITC	50	+++	+++	+
	100	+++	+++	+
	150	+++	++	-
	200	+++	++	-
BITC	50	+++	+++	+
	100	+++	+++	+
	150	+++	++	+
	200	+++	++	-
MITC	50	+++	+++	+
	100	+++	++	+
	150	+++	++	-
	200	+++	++	-
PEITC	50	+++	+++	+
	100	+++	+++	+
	150	+++	+++	+
	200	+++	+++	+

Symbols indicate the degree of growth as determined by colony size. Symbols indicate the colony size compared to the control: +++ indicates greater than 90%, ++ indicates 50–90%, + indicates less than 50%, and - indicates no growth.
